# Increasing Disadvantages in Cancer Survival in New Zealand Compared to Australia, between 2000-05 and 2006-10

**DOI:** 10.1371/journal.pone.0150734

**Published:** 2016-03-03

**Authors:** J. Mark Elwood, Phyu Sin Aye, Sandar Tin Tin

**Affiliations:** Section of Epidemiology and Biostatistics, School of Population Health, University of Auckland, Auckland, New Zealand; University of Sydney, AUSTRALIA

## Abstract

New Zealand has lower cancer survival compared to its neighbour Australia. If this were due to long established differences between the two patient populations, it might be expected to be either constant in time, or decreasing, as improving health services deals with inequities. In this study we compared trends in relative cancer survival ratios in New Zealand and Australia between 2000–05 and 2006–10, using data from the New Zealand Cancer Registry and the Australian Institute for Health and Welfare. Over this period, Australia showed significant improvements (6.0% in men, 3.0% in women) in overall 5-year cancer survival, with substantial increases in survival from major cancer sites such as lung, bowel, prostate, and breast cancers. New Zealand had only a 1.8% increase in cancer survival in men and 1.3% in women, with non-significant changes in survival from lung and bowel cancers, although there were increases in survival from prostate and breast cancers. For all cancers combined, and for lung and bowel cancer, the improvements in survival and the greater improvements in Australia were mainly in 1-year survival, suggesting factors related to diagnosis and presentation. For breast cancer, the improvements were similar in each country and seen in survival after the first year. The findings underscore the need to accelerate the efforts to improve early diagnosis and optimum treatment for New Zealand cancer patients to catch up with the progress in Australia.

## Introduction

Cancer survival has been recognised as a key indicator for monitoring and assessment of cancer care effectiveness [1–3]. Generally, if the quality of cancer care is comparable and background health status is similar, survival outcomes for patient populations with a specific cancer should be similar for a specific period, regardless of geographical, ethnic or socioeconomic variations [1–3]. However, many studies, including those of EUROCARE [4–6], CONCORD [7,8], the International Cancer Benchmarking Partnership (ICBP) [9], and the Organisation for Economic Cooperation and Development (OECD) [10], found wide variations in cancer survival and access to optimal cancer care internationally [9–12].

New Zealand and Australia were positioned among the countries with relatively high cancer survival in the CONCORD-2 [8] study but survival rates in New Zealand were generally inferior to its neighbour Australia despite similar health systems, professional standards and treatment guidelines for cancer patients. Australia ranked well in an earlier CONCORD international comparison with survival outcomes comparable to those of the US, Japan and certain European countries [7].

Our previous work showed that during 2006–10, the 5-year relative survival ratios (RSRs) for all cancers combined in New Zealand were 3.8% lower for men and 4.2% lower for women than those in Australia [13]. From this difference we estimated that about 12% of deaths from cancer in 5 years after diagnosis would be avoided if cancer survival in New Zealand were the same as that in Australia during this period [13], which is consistent with an estimate using slightly different methods[14], and is consistent with the differences seen in total population mortality [15]. These findings suggest that New Zealand has room for improvement in cancer care.

In both New Zealand and Australia, discrepancies in cancer survival exist within the country with regard to ethnicity, geography, and socioeconomic position. Studies in New Zealand show that indigenous Māori and Pacific people have lower cancer survival, which is partially attributable to geographical factors, stage at diagnosis, waiting time from diagnosis to treatment and timeliness for curative treatment [16–23]. Similarly in Australia, disparities in cancer survival are seen among different ethnic groups with indigenous people being at a disadvantage, and are shown to be associated with geographical factors and socioeconomic variations [24–30].

If the difference in cancer survival between New Zealand and Australia in the recent study were due to long established differences between the two patient populations, for example, in ethnic, social, or geographical factors, it might be expected to be either constant in time, or decreasing, as improving health services deal with some of these inequities. Therefore we asked whether over a recent time period, the disadvantages in cancer survival ratios in New Zealand compared with Australia have decreased, continued, or increased.

## Materials and Methods

The survival of cancer patients was estimated using RSRs annually, beginning at 1 year after diagnosis up to 10 years, with their corresponding 95% confidence intervals (95% CIs). The RSR is the ratio of the observed survival rate in the patient group to the expected survival rate in the group of individuals from the general population which is similar to the patient group with respect to age, gender, race, and calendar period of observation [31,32]. The expected survivals were derived by Ederer II method [33,34].

Survival data for Australia were obtained from the Australian Institute for Health and Welfare (AIHW) [35]. New Zealand data were obtained from the New Zealand Cancer Registry (NZCR) [36]. The data cover all primary cancers excluding basal cell and squamous cell carcinoma of the skin, and are recorded with the standard ICD-10 coding system. Both countries have similar coverage and quality control of data. The RSRs were estimated using a period approach for the whole population in each country, by cancer site and gender of patients who were diagnosed in 2000–05 and 2006–10. Age adjustment was not undertaken in the survival analyses as the age distributions for all cancers combined and for the most common cancers were found to be virtually identical between the two countries.

Changes in the gender-specific RSRs at 1, 5, and 10 years in each country over time were calculated by subtracting the RSRs in 2000–05 from the RSRs in 2006–10, for each cancer site. Differences in the RSR changes between the two countries were then calculated. A z-test [31] was used to compare the RSRs at the two periods in each country and to compare changes in the RSRs between the two countries. Only 5-year RSRs and those cancers having more than 100 deaths per year are presented, and significant differences are underlined and bolded. One- and 10-year results are given in S1 Table.

### Ethics statement

Ethics approval was not required as completely de-identified data were used for all analyses.

## Results

### Cancer survival changes between 2000–05 and 2006–10 in Australia

**Fig 1:**
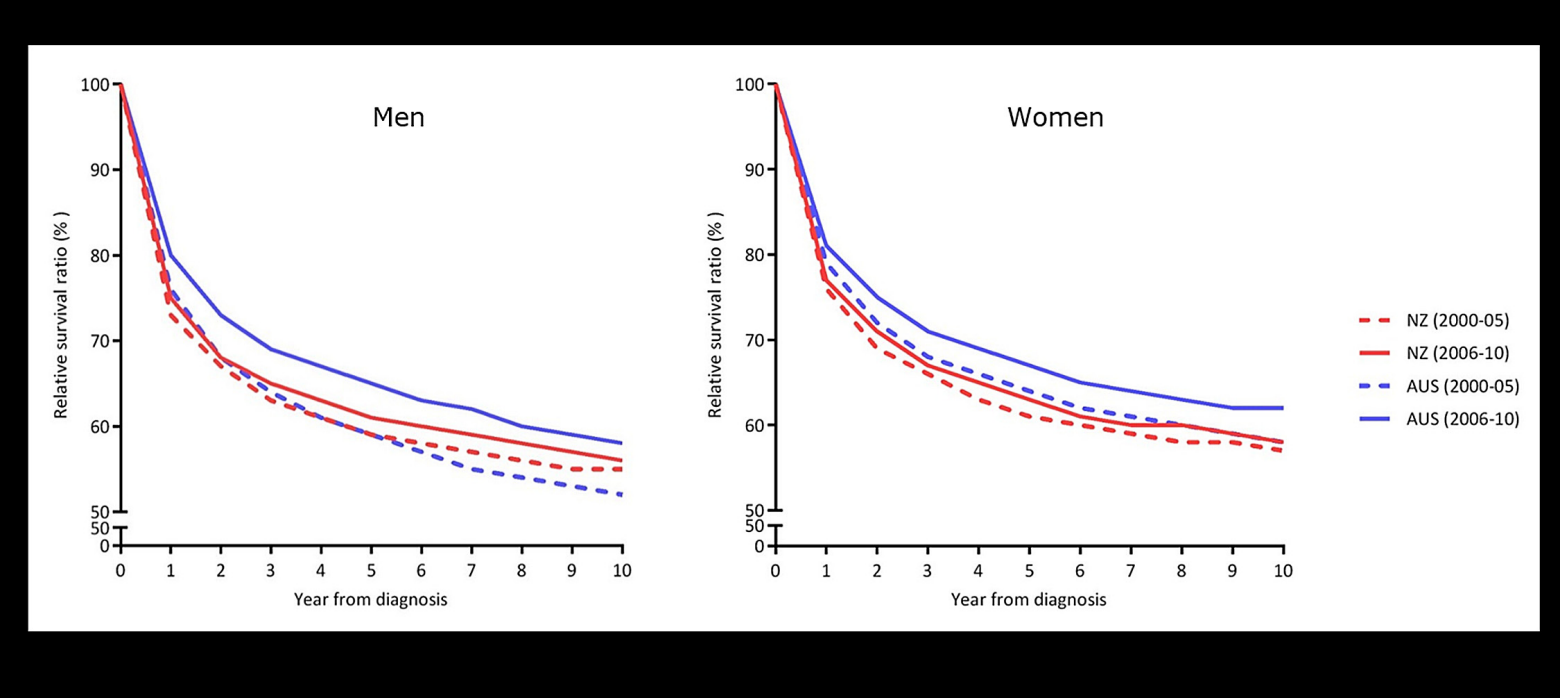
Relative survival ratios for all cancers combined comparing New Zealand and Australia, 2000–2005 and 2006–2010 (men and women).

In Australia there was a substantial improvement in survival from all cancers combined from 2000–05 and 2006–10 showing a 6.0% (59.1% to 65.1%) increase in 5-year RSR in men and a 3.0% (64.4% to 67.4%), in women (Fig 1 and Table 1).

**Table 1:** Changes in cancer 5-year relative survival ratios over time (2000–05 and 2006–10) and comparison of changes in New Zealand and Australia

Cancer site^a^	Sex	NZ^b^ annual deaths 2008	5-year RSR NZ^b^(95% CI) [2000–05]	Improve NZ^b^ (95% CI) [2000–05–2006–10]	5-year RSR AUS^c^ (95% CI) [2000–05]	Improve AUS^c^ (95% CI) [2000–05–2006–10]	Difference (95% CI) [Improve AUS^c^ - Improve NZ^b^]
**All cancer**	M	4561	59.54 (59.02, 60.06)	**1.80** (1.06, 2.54)	59.10 (58.90, 59.30)	**6.00** (5.68, 6.32)	**4.20** (3.39, 5.01)
	F	4005	61.92 (61.42, 62.42)	**1.25** (0.53, 1.97)	64.40 (64.20, 64.60)	**3.00** (2.68, 3.32)	**1.75** (0.96, 2.54)
**Lung**	M	889	7.62 (6.91, 8.37)	0.91 (-0.19, 2.01)	10.80 (10.50, 11.20)	**1.80** (1.19, 2.41)	0.89 (-0.37, 2.15)
	F	745	10.14 (9.17, 11.17)	0.41 (-1.00, 1.82)	14.40 (13.80, 15.00)	**2.10** (1.18, 3.02)	**1.69** (0.00, 3.38)
**Bowel**	M	684	59.84 (58.42, 61.25)	0.51 (-1.52, 2.54)	61.70 (61.10, 62.30)	**3.60** (2.68, 4.52)	**3.09** (0.86, 5.32)
	F	580	61.25 (59.85, 62.63)	0.96 (-1.07, 2.99)	62.50 (61.80, 63.10)	**4.60** (3.61, 5.59)	**3.64** (1.38, 5.90)
**Prostate**	M	670	87.42 (86.48, 88.34)	**2.91** (1.64, 4.18)	85.30 (84.90, 85.70)	**6.70** (6.17, 7.23)	**3.79** (2.41, 5.17)
**Breast**	F	618	83.73 (82.91, 84.53)	**2.91** (1.79, 4.03)	87.40 (87.10, 87.70)	**2.00** (1.54, 2.46)	-0.91 (-2.13, 0.31)
**Pancreas**	M	176	4.09 (3.05, 5.35)	0.58 (-1.16, 2.32)	5.30 (4.70, 5.90)	-0.40 (-1.28, 0.48)	-0.98 (-2.93, 0.97)
	F	197	3.37 (2.38, 4.62)	0.95 (-0.69, 2.59)	4.90 (4.30, 5.50)	0.70 (-0.26, 1.66)	-0.25 (-2.15, 1.65)
**Melanoma**	M	202	86.84 (85.39, 88.23)	1.31 (-0.65, 3.27)	89.60 (89.00, 90.10)	**-1.10** (-1.91, -0.29)	**-2.41** (-4.53, -0.29)
	F	115	93.28 (92.11, 94.39)	0.50 (-1.12, 2.12)	94.00 (93.60, 94.50)	-0.40 (-1.15, 0.35)	-0.9 (-2.68, 0.88)
**NHL**	M	160	55.35 (52.64, 58.01)	**9.88** (6.14, 13.62)	62.00 (60.90, 63.10)	**8.00** (6.33, 9.67)	-1.88 (-5.98, 2.22)
	F	134	57.87 (55.05, 60.63)	**6.14** (2.18, 10.10)	62.60 (61.50, 63.70)	**8.70** (6.96, 10.44)	2.56 (-1.77, 6.89)
**Stomach**	M	173	18.61 (16.41, 20.94)	**5.58** (1.99, 9.17)	24.00 (22.90, 25.10)	**2.90** (1.04, 4.76)	-2.68 (-6.72, 1.36)
	F	110	20.61 (17.70, 23.70)	2.17 (-2.41, 6.75)	25.70 (24.20, 27.20)	0.70 (-1.80, 3.20)	-1.47 (-6.69, 3.75)
**Oesophagus**	M	154	9.74 (7.72, 12.06)	0.55 (-2.57, 3.67)	16.20 (15.00, 17.50)	-0.70 (-2.58, 1.18)	-1.25 (-4.89, 2.39)
	F	75	11.87 (8.91, 15.33)	-0.91 (-5.45, 3.63)	18.50 (16.80, 20.30)	-1.50 (-4.31, 1.31)	-0.59 (-5.93, 4.75)
**Brain**	M	109	17.82 (15.42, 20.38)	0.68 (-3.13, 4.49)	18.70 (17.60, 19.90)	1.70 (-0.19, 3.59)	1.02 (-3.23, 5.27)
	F	98	20.92 (17.81, 24.21)	2.14 (-2.63, 6.91)	20.30 (19.00, 21.70)	**3.60** (1.23, 5.97)	1.46 (-3.87, 6.79)
**Bladder**	M	134	70.26 (67.64, 72.83)	**-17.16** (-21.29, -13.03)	62.40 (61.20, 63.60)	**-2.40** (-4.40, -0.40)	**14.76** (10.17, 19.35)
	F	66	64.38 (60.19, 68.43)	**-18.64** (-25.15, -12.13)	54.00 (52.10, 56.00)	**-4.40** (-7.73, -1.07)	**14.24** (6.93, 21.55)
**Liver**	M	124	8.00 (6.08, 10.26)	**4.91** (1.59, 8.23)	12.20 (11.00, 13.30)	**3.30** (1.49, 5.11)	-1.61 (-5.39, 2.17)
	F	66	12.69 (9.11, 16.92)	0.45 (-4.76, 5.66)	12.50 (10.70, 14.40)	2.90 (-0.05, 5.85)	2.45 (-3.54, 8.44)
**Ovary**	F	184	45.56 (43.03, 48.07)	**-9.71** (-13.37, -6.05)	40.20 (39.00, 41.50)	**3.10** (1.11, 5.09)	**12.81** (8.65, 16.97)
**Kidney**	M	98	57.50 (54.17, 60.75)	**4.73** (0.18, 9.28)	66.10 (64.80, 67.40)	**5.50** (3.55, 7.45)	0.77 (-4.18, 5.72)
	F	67	57.00 (52.90, 60.96)	**10.42** (4.72, 16.12)	66.40 (64.70, 68.00)	**6.10** (3.51, 8.69)	-4.32 (-10.58, 1.94)
**Myeloma**	M	96	35.52 (31.59, 39.54)	**7.96** (2.13, 13.79)	36.30 (34.50, 38.10)	**7.60** (4.76, 10.44)	-0.36 (-6.85, 6.13)
	F	68	34.47 (30.06, 39.00)	4.56 (-1.89, 11.01)	36.40 (34.40, 38.40)	**6.40** (3.28, 9.52)	1.84 (-5.33, 9.01)

^a^ Cancer sites are ordered by annual number of deaths in NZ in 2008, and only those accounted for more than 100 deaths are included in the table.

^b^ NZ = New Zealand

^c^ AUS = Australia

As shown in Table 1, there were significant improvements in 5-year RSRs for almost all major cancer sites: lung (1.8% increase in men, and 2.1% increase in women), bowel (3.6% increase in men, and 4.6% increase in women), prostate (6.7% increase), and breast (2**.0**% increase).

The other cancer sites which had significant improvements in 5-year RSRs were, in both sexes, non-Hodgkin lymphoma (NHL, men 8.0%, women 8.7%), kidney (men 5.5%, women 6.1%), myeloma (men 7.6%, women 6.4%), and also, in men only, stomach (2.9%) and liver (3.3%); and in women only, brain (3.6%), and ovary (3.1%).

Bladder cancer in both men and women showed a statistically significant decrease in 5-year RSRs; a 2.4% decline in men and 4.4% decline in women. Also, melanoma showed a significant 1.1% decline in men, from a survival ratio of 89.6% in 2000–05.

Other cancers showed no significant change in 5-year RSRs between these two time periods.

### Cancer survival changes between 2000–05 and 2006–10 in New Zealand

In New Zealand, there was a modest, but statistically significant, improvement from 2000–2005 to 2006–2010 for all cancers combined, showing a 1.8% increase (59.5% to 61.3%) in 5-year RSRs in men and a 1.3% increase (61.9% to 63.2%) in women (Fig 1 and Table 1).

As shown in Table 1, among major cancers, prostate and breast cancers had significant improvements over time, each showing a 2.9% increase in 5-year RSRs, whereas lung cancer and bowel cancer showed only very small (less than 1%) increases, which were not statistically significant.

The other cancer sites which had significant improvements in 5-year RSRs were, in both men and women, non-Hodgkin lymphoma (NHL) (men 9.9% and women 6.1% increase,), kidney cancer (men 4.7%, women 10.4% increase), and also, but in men only, stomach cancer (5.6% increase), liver cancer (4.9% increase), and myeloma (8.0% increase).

For bladder cancer, there was a large and significant decrease in 5-year RSRs, showing a 17.2% decline in men and 18.6% decline in women. For ovarian cancer, survival also reduced, by 9.7%.

Other cancers showed no significant change in 5 year survival over time.

### Comparing cancer survival changes between New Zealand and Australia

When cancer survival changes from 2000–05 to 2006–10 were compared between the two countries, the improvements in RSRs were significantly larger in Australia than in New Zealand, for all cancers combined in each gender (Fig 1 and Table 1).

As shown in Table 1, for major cancers, Australia had significantly larger improvements than New Zealand in 5-year RSRs for lung cancer (in men), bowel cancer (in both genders), and prostate cancer.

For bladder cancer, survival decreased (worsened) in both countries, but the decrease was much larger in New Zealand, in both genders. For ovarian cancer, New Zealand showed a decrease of 9.7% while Australia showed an improvement of 3.1%, resulting in 12.8% difference in RSR changes.

The only site for which New Zealand had a significantly better survival trend was melanoma in men, where in Australia survival decreased by 1.1% from a very high value of 89.6% in the earlier time period, while in New Zealand survival increased slightly from a lower ratio of 86.8%.

For other cancers the differences in survival changes between the two countries were not statistically significant.

## Discussion

This study found that there was a substantial difference in improvements in cancer survival over time between New Zealand and Australia. From 2000–05 to 2006–2010, Australia showed significant improvements (6% in men, 3% in women) in overall cancer survival, contributed to by substantial increases in survival from common cancers such as lung, bowel, prostate, and breast cancers. For the same period, New Zealand had only a 1.8% increase in cancer survival in men and 1.3% in women, with non-significant changes in survival from lung and bowel cancers, although there were some increases in survival from prostate and breast cancers. For all cancers combined, the 5-year RSR in Australia increased, compared to New Zealand, by about 4.2% in men and 1.8% in women in the 5 year period between patients diagnosed in 2000–05 and 2006–10.

In comparing cancer survival among different populations, comparability issues such as those related to data, tumour and host (patient) should be considered carefully [37]. Data comparability issues in interpreting survival differences have been discussed in the EUROCARE and CONCORD international cancer survival comparison studies [6,7,38,39]. This analysis used the data from the cancer registries which cover the entire population in both New Zealand and Australia, and similar methods were used for cancer coding, linkage, analysis, and maintenance of data quality [35,36]. Differences in data collection methods could contribute to variations in survival due to a lead time effect, with increased survival contributed to by an earlier date of diagnosis without affecting the date of death [40,41], but our inquiries did not suggest this in the registries used. The survival differences found are supported by previous studies on cancer mortality comparisons, which showed substantially higher cancer mortality in New Zealand compared to Australia with little change over time [15,42,43]. We therefore believe that data comparability issues do not explain the survival differences.

However, differences between registries in the timing of changes in procedures may account for trends for sites that have shown decreases in cancer survival: bladder cancer in both countries, and ovarian cancer in New Zealand. For bladder cancer, changes made in the WHO grading system of bladder cancer in 2004 [44,45] reassigned some low malignancy tumours from being previously grade-1 tumours to the classification of ‘papillary urothelial neoplasm of low malignant potential (PUNLMP)’ and exclusion of these from survival analyses would produce lower survival. The EUROCARE studies noted: “Previous EUROCARE studies have documented marked differences in survival for bladder cancer across European populations. However, the interpretation of these survival differences is problematic because criteria for establishing the invasiveness of lesions in the urothelium are not well standardised.[6]” There is little evidence of ethnic disparity for bladder cancer in New Zealand [23].

For ovarian cancer, the New Zealand registry ceased to include ovarian cancers of borderline malignancy from 1 January 2003, causing a similar decrease in survival of included cancers [46]; however, Australian registries made a similar change [47].

We found smaller survival improvements in New Zealand for many cancer sites including the most common cancers such as lung, bowel, and prostate cancers. For host factors, we examined the age distribution for all cancers combined and each of the most common cancers, and found it to be virtually identical. Also, we did analyses for men and women separately. While comorbidity is common in cancer patients and might be related to cancer outcomes [48–50], survival outcomes do not depend on longevity and cause of death as the RSRs of cancer patients are estimated in comparison to similar year-age-sex groups in the general population.

In our previous study comparing survival in Australia and New Zealand in 2006–10[13], the higher survival in Australia for many cancers was seen at 1 year, with little difference in conditional survival from 1 to 5 years, and from 5 to 10 years. This suggests differences related to diagnosis and initial presentation rather than later treatment, as noted in European and world-wide comparisons [6,7]; these in turn relate to awareness of symptoms, and to primary care management in referral, investigation, and diagnosis. The International Cancer Benchmarking Project (ICBP) has shown that international variations in primary care processes are associated with differences in cancer survival [51]. Two Australian states (Victoria and New South Wales) were involved in that study, showing generally good results for access to investigations, wait times for tests, and primary care physicians’ responses to clinical vignettes as compared to regions in the UK, continental Europe and Canada. A New Zealand study using identical methods has now been done (Htun, Elwood et al., in preparation) showing that in comparison to Victoria and New South Wales, primary care physicians’ (PCP) access to tests and to specialist advice in New Zealand was more limited and wait times for testing were longer; for example, 41% of New Zealand PCPs reported that they could get a referral for a suspected cancer patient within 48 hrs, compared to 60% and 59% in the Australian regions; average times for a colonoscopy to be done and reported were 20 weeks in New Zealand, compared to 5–6 weeks in Australia. The ICBP also assessed cancer awareness and beliefs in Canada, Europe and Australia (again Victoria and New South Wales) [52]; barriers to symptomatic presentation were reported as lower in Australia than in the UK, but there was no association with 1-year survival rates. New Zealand was not included in this survey.

In the current analysis, the increasing advantage for Australia over time was greatest in 1 year survival, but also applied throughout the follow up period: compared to New Zealand, 1 year survival, 1 to 5 year conditional survival and 5 to 10 year conditional survival improved more in Australia by 2.3, 1.9, and 1.1% for all cancer in males, and 1.1, 0.6, and 0.5% in females (derived from S1 Table). While this pattern could be due to differences in staging at diagnosis, it could also suggest that care throughout the follow up period is better in Australia. This pattern varied between cancer sites: lung and colorectal showed the main differences in 1 year survival, while for prostate the Australian advantage was seen in later survival.

Differences in cancer survival trends are, therefore, most likely to be due to health care related factors such as early diagnosis and optimum treatment. Australia took major national-level actions for better cancer care earlier than New Zealand. The Australian federal government identified cancer control as one of the nine National Health Priority areas in 1996 [53,54], and supported the National Breast Cancer Centre (NBCC) from 1995, the National Cancer Control Initiative from 1997, and subsequently Cancer Australia (incorporating the NBCC) from 2006. Cancer Australia works collaboratively with various stakeholders and cancer care service providers to provide comprehensive cancer prevention, diagnosis, treatment, and research, including an emphasis on disadvantaged indigenous cancer patients [55]. All these groups had substantial commitment from non-government sectors, particularly the Cancer Council Australia and the Councils in each state, and the Clinical Oncology Society of Australia, representing cancer specialists. Such collaboration produced an influential report on optimising cancer care in 2003[56]. This emphasised, among many issues, multidisciplinary care and patient representation at all levels, drawing on work pioneered in breast cancer [57]. The accelerating national level actions for improvement of cancer care services may have underpinned the better cancer survival outcomes in Australia.

The New Zealand Cancer Control Strategy Action Plan began in 2005–2010 aiming to reduce the cancer incidence, impact of cancer and inequalities regarding cancer [58]. Starting from 2008, the Ministry of Health has led a national work programme for cancer care improvements [59]. The Ministry of Health made recommendations on disparity and access to care in 2009 to facilitate primary care practitioners for a timely referral of cancer suspected patients for further management [60]. These national efforts, however, have been less ambitious than those in Australia.

It is well known that ethnic and socioeconomic inequities exist in health care and outcomes. Indigenous people have poorer general health status than non-indigenous people in both countries. The difference is more marked in Australia; while indigenous people in New Zealand have a life expectancy nine years lower than the non-indigenous majority, the difference is more than nineteen years in Australia [61]. In response, the Australian Government established the Primary Health Care Access Program (PHCAP) in 2001 and set out a national implementation to improve indigenous access and control over primary health care services [61,62].

Australia has undertaken clinical management and population-based cancer care assessment studies [63] to a greater extent than has New Zealand. These may act as triggers for improvement in cancer care, with a focus on inequity issues and on adherence to clinical guidelines. Robust assessments of cancer care and provisions of comprehensive information for improving health services are still relatively limited in New Zealand.

Both New Zealand and Australia have national agencies for the approval and the public-sector funding of drugs; budget limitations are stricter in New Zealand [64] and new drug approvals often slower. Documentation of differences over time is beyond the scope of this paper, but in 2015, 88 cancer drugs were funded in both countries, although not necessarily for the same indications, and 22 were funded in Australia but not in New Zealand. Of these, ten had evidence of a gain in overall survival above a ‘low’ boundary for clinically meaningful gain [64], and two were above the ‘high’ boundary. The New Zealand agency concludes that health gains from most of these extra drugs would be marginal, but oncologists and advocacy groups have challenged this [65]. In relation to this, investment in health research and development, including clinical trials, is lower in New Zealand: government financed being 0.5% of gross domestic product and business financed 0.47%, compared to 0.77% and 1.36% respectively in Australia [66]. The participation of cancer patients in trials seems likely to be lower in New Zealand.

In New Zealand, mortality inequalities among different ethnic and income groups became stable or narrowed in New Zealand from 1996–1999 to 2001–2004 [67] but cancer is still an important contributor to ethnic and income inequalities in mortality [67,68]. A national cancer survival analysis concluded that between 1991 and 2004 there was evidence for ethnic and income differences in survival, with little change over time, from most cancers including lung, bowel and female breast. Income differences were also seen for prostate cancer [23]. The New Zealand Government has been making efforts to improve cancer care and address inequities.

We hypothesised that if such cancer care efforts were effective in both countries, the gap in survival outcomes between the two countries would remain constant or become narrower over time. However, this study shows that the differences are increasing. This underscores the need for more efforts to improve cancer care and reduce disparities in New Zealand. As more avoidable deaths can be saved from small gains in survival for common cancers rather than large gains for uncommon cancers [69], it is important for cancer care services to give priority on common cancers such as lung, bowel, prostate, and female breast.

Lung cancer has been the leading cause of death among major cancers with very low survival rates. Australia showed modest but significant improvements in survival from lung cancer over time, whereas New Zealand had little improvement; the difference in progress between the countries was greater in women. This coincides with a greater difference in prevalence of tobacco smoking for women than men–the prevalences being higher in New Zealand than Australia [70]. Additionally, substantial ethnic disparities exist in New Zealand, with Maori having a higher risk of having advanced disease and a lower chance of receiving curative treatment compared to non-Maori [18, 71–73]. In one large region in New Zealand, 36% of lung cancer patients first presented at an emergency department [74], and in stage 1 and 2 non-small cell lung cancer, fewer patients were treated with curative intent, and fewer within recommended time frames, than in Australia or other countries [75].

Survival from bowel cancer improved significantly in Australia, in both sexes, but not in New Zealand. In 2000–2004, from registry data, 28% of colorectal cancer was diagnosed at a localised stage in New Zealand, compared to 34% in New South Wales [76]. In Australia a national population-based bowel cancer screening program using an immunochemical test was introduced in 2006 [77, 78], although implementation is gradual and a second biennial test will not be offered to all aged 50–74 until 2020[79]. Although this national program has probably not affected survival rates yet, it was preceded by many years of attention to the problem and by localised programs. In New Zealand, a four year bowel cancer screening pilot in one region began in 2011 [80]. An audit and research program, the PIPER project (Presentation, Investigation, Pathways, Evaluation, Rx (treatment)), including over 6000 bowel cancer patients in New Zealand, may indicate priorities for improvement[81]. Ethnic disparities for bowel cancer in New Zealand also remain large, with Maori having a 20% lower survival compared to non-Maori during 1994–2007 [46].

Improvement in survival from prostate cancer was better in Australia than in New Zealand. However, it is difficult to interpret this as the incidence and survival of prostate cancer vary widely and are greatly affected by over-diagnosis through screening for Prostate Specific Antigen [6, 15].

Survival from breast cancer improved by a similar amount in the two countries. This may show comparable progress in early diagnosis and treatment, as this cancer has received the most attention. Both countries have had free population-based mammographic screening programmes for many years, with continued efforts to improve participation. Participation in the national programmes has been higher in New Zealand; in 2008–10, 69% at ages 50–69 compared to 55% in Australia [82], although there may be more out-of-programme screening in Australia. Breast cancer is the one major cancer where Australian survival in 2006–10 was only slightly higher at 1 year, but the differences became larger at 5 and 10 years [13], suggesting that diagnosis may be comparable in the two countries but differences in further treatment may be important. In the present analysis, the improvements over time in both countries were also greater for survival after the first year. Reductions in breast cancer mortality in Australia have been associated with the increased use of adjuvant hormonal and chemo-therapy [83]. In New Zealand, ethnic disparities between Maori and non-Maori have reduced over time [84]. Surgeons in both countries contribute to audit and improvement processes [85].

The most common haematological malignancies such as non-Hodgkin lymphoma (NHL) and myeloma showed substantial survival improvements in both countries, although non-significant for myeloma in New Zealand women. These improvements in survival are likely due to advanced treatments such as stem cell transplantation and immune-modulatory drug therapies [86–89]. In addition, the survival improvement for NHL has been associated with a decrease in the proportion of HIV-related NHL due to effective anti-retroviral therapy [89].

Among cancers that are responsible for more than 100 deaths annually, only survival from melanoma improved significantly in New Zealand compared to Australia. Survival ratios are very high in both countries, and there are many tumours assessed as in-situ that would be excluded from registries [90]—a small change in pathology practice could explain the small decrease in survival seen in Australia.

In conclusion, lower improvements in cancer survival in New Zealand compared to Australia over time are likely to indicate persistent or increasing disparities in cancer care services between the two countries. Health care policies and clinical improvement efforts have been more ambitious and comprehensive in Australia. This emphasises the need to accelerate the efforts to improve early diagnosis and optimum treatment for New Zealand cancer patients to catch up with the progress in Australia. As ethnic disparities remain and are attributable to substantial differences in cancer outcomes, research on access to cancer diagnosis and treatment services across different ethnic groups would be beneficial to provide specific recommendations towards better overall cancer outcomes in the future.

## Supporting Information

S1 Table 1, 5, and 10 year relative survival ratio changes over time and Australia-New Zealand comparisons (PDF)
