# Supplementary material for: Increasing Disadvantages in Cancer Survival in New Zealand Compared to Australia, between 2000-05 and 2006-10
Source: PLoS One. 2016 Mar 3;11(3):e0150734. doi: 10.1371/journal.pone.0150734 (PMC4777383; doi:10.1371/journal.pone.0150734)
Supplement: S1 Table — (PDF) [file pone.0150734.s001.pdf]

S1 Table. 1, 5, and 10 year relative survival ratio changes over time and Australia-New Zealand comparisons

Cancer site	Sex	NZ annual deaths (2008)	Year	RSR NZ (2000-05)	95%CI of RSR NZ (2000-05)		Imp NZ (2000-05 to 2006-10)	95%CI of Imp NZ (2000-05 to 2006-10)		RSR AUS (2000-05)	95%CI of RSR AUS (2000-05)		Imp AUS (2000-05 to 2006-10)	95%CI of Imp AUS (2000-05 to 2006-10)		Difference (Imp AUS - Imp NZ)	95%CI of Difference	
**All cancer**	M	4561	*1*	73.79	73.40	74.18	**1.57**	1.01	2.13	76.10	76.00	76.30	**3.90**	3.61	4.19	**2.33**	1.70	2.96
			5	59.54	59.02	60.06	**1.80**	1.06	2.54	59.10	58.90	59.30	**6.00**	5.68	6.32	**4.20**	3.39	5.01
			10	55.40	54.54	56.26	**1.20**	0.12	2.27	52.20	51.90	52.40	**6.50**	6.11	6.89	**5.30**	4.16	6.44
	F	4005	*1*	76.10	75.70	76.50	**1.05**	0.48	1.62	79.30	79.10	79.50	**2.20**	1.88	2.52	**1.15**	0.50	1.80
			5	61.92	61.42	62.42	**1.25**	0.53	1.97	64.40	64.20	64.60	**3.00**	2.68	3.32	**1.75**	0.96	2.54
			10	57.39	56.62	58.15	**1.32**	0.35	2.29	58.50	58.20	58.70	**3.60**	3.21	3.99	**2.28**	1.23	3.33
**Lung**	M	889	*1*	25.19	24.10	26.29	0.65	-0.97	2.27	34.40	33.90	34.90	**1.60**	0.66	2.54	0.95	-0.93	2.83
			5	7.62	6.91	8.37	0.91	-0.19	2.01	10.80	10.50	11.20	**1.80**	1.19	2.41	0.89	-0.37	2.15
			10	6.48	5.62	7.42	0.52	-0.73	1.77	7.70	7.30	8.00	**1.10**	0.53	1.67	0.58	-0.79	1.95
	F	745	*1*	30.06	28.68	31.45	**1.99**	0.01	3.97	39.50	38.70	40.20	**2.90**	1.61	4.19	0.91	-1.45	3.27
			5	10.14	9.17	11.17	0.41	-1.00	1.82	14.40	13.80	15.00	**2.10**	1.18	3.02	**1.69**	0.00	3.38
			10	8.30	7.14	9.57	-0.03	-1.60	1.54	10.30	9.70	10.80	**2.00**	1.15	2.85	**2.03**	0.24	3.82
**Bowel**	M	684	*1*	79.04	78.02	80.03	0.65	-0.79	2.09	82.00	81.60	82.40	**3.50**	2.82	4.18	**2.85**	1.26	4.44
			5	59.84	58.42	61.25	0.51	-1.52	2.54	61.70	61.10	62.30	**3.60**	2.68	4.52	**3.09**	0.86	5.32
			10	57.50	55.24	59.76	-2.06	-4.97	0.85	55.30	54.60	56.10	**3.80**	2.70	4.90	**5.86**	2.75	8.97
	F	580	*1*	77.97	76.93	78.97	0.25	-1.24	1.74	80.60	80.10	81.10	**3.70**	2.88	4.52	**3.45**	1.75	5.15
			5	61.25	59.85	62.63	0.96	-1.07	2.99	62.50	61.80	63.10	**4.60**	3.61	5.59	**3.64**	1.38	5.90
			10	57.67	55.32	60.03	0.92	-2.07	3.91	57.80	57.00	58.60	**4.50**	3.26	5.74	**3.58**	0.34	6.82
**Prostate**	M	670	*1*	95.81	95.36	96.24	**0.70**	0.09	1.31	96.10	95.90	96.30	**1.70**	1.42	1.98	**1.00**	0.33	1.67
			5	87.42	86.48	88.34	**2.91**	1.64	4.18	85.30	84.90	85.70	**6.70**	6.17	7.23	**3.79**	2.41	5.17
			10	80.54	78.31	82.75	**4.47**	1.88	7.06	75.10	74.50	75.70	**9.40**	8.55	10.25	**4.93**	2.20	7.66
**Breast**	F	618	*1*	96.30	95.90	96.68	**0.89**	0.36	1.42	97.30	97.20	97.50	**0.60**	0.31	0.89	-0.29	-0.90	0.32
			5	83.73	82.91	84.53	**2.91**	1.79	4.03	87.40	87.10	87.70	**2.00**	1.54	2.46	-0.91	-2.13	0.31
			10	75.61	74.02	77.16	**4.06**	2.16	5.96	79.30	78.90	79.80	**3.90**	3.26	4.54	-0.16	-2.16	1.84

Cancer site	Sex	NZ annual deaths (2008)	Year	RSR NZ (2000-05)	95%CI of RSR NZ (2000-05)		Imp NZ (2000-05 to 2006-10)	95%CI of Imp NZ (2000-05 to 2006-10)		RSR AUS (2000-05)	95%CI of RSR AUS (2000-05)		Imp AUS (2000-05 to 2006-10)	95%CI of Imp AUS (2000-05 to 2006-10)		Difference (Imp AUS - Imp NZ)	95%CI of Difference	
**Pancreas**	M	176	*1*	12.37	10.63	14.25	1.70	-0.92	4.32	20.50	19.60	21.50	**2.30**	0.57	4.03	0.60	-2.54	3.74
			5	4.09	3.05	5.35	0.58	-1.16	2.32	5.30	4.70	5.90	-0.40	-1.28	0.48	-0.98	-2.93	0.97
			10	4.30	3.13	5.74	0.29	-1.67	2.25	4.30	3.70	5.00	-0.60	-1.48	0.28	-0.89	-3.04	1.26
	F	197	*1*	11.50	9.85	13.29	1.09	-1.36	3.54	20.10	19.10	21.10	0.40	-1.40	2.20	-0.69	-3.73	2.35
			5	3.37	2.38	4.62	0.95	-0.69	2.59	4.90	4.30	5.50	0.70	-0.26	1.66	-0.25	-2.15	1.65
			10	2.78	1.71	4.30	1.43	-0.43	3.29	3.90	3.30	4.50	0.30	-0.62	1.22	-1.13	-3.20	0.94
**Melanoma**	M	202	*1*	95.50	94.75	96.19	0.84	-0.14	1.82	97.00	96.80	97.30	**-0.60**	-1.07	-0.13	**-1.44**	-2.53	-0.35
			5	86.84	85.39	88.23	1.31	-0.65	3.27	89.60	89.00	90.10	**-1.10**	-1.91	-0.29	**-2.41**	-4.53	-0.29
			10	85.35	82.91	87.72	-0.24	-3.28	2.80	85.60	84.80	86.30	-0.40	-1.46	0.66	-0.16	-3.38	3.06
	F	115	*1*	98.11	97.53	98.61	0.24	-0.52	1.00	98.40	98.20	98.60	-0.10	-0.50	0.30	-0.34	-1.20	0.52
			5	93.28	92.11	94.39	0.50	-1.12	2.12	94.00	93.60	94.50	-0.40	-1.15	0.35	-0.90	-2.68	0.88
			10	92.60	90.56	94.54	0.27	-2.27	2.81	91.80	91.10	92.40	-0.40	-1.39	0.59	-0.67	-3.40	2.06
**NHL**	M	160	*1*	74.65	72.49	76.69	**4.93**	2.08	7.78	79.50	78.70	80.30	**4.20**	2.88	5.52	-0.73	-3.87	2.41
			5	55.35	52.64	58.01	**9.88**	6.14	13.62	62.00	60.90	63.10	**8.00**	6.33	9.67	-1.88	-5.98	2.22
			10	47.15	43.29	51.02	**9.51**	4.43	14.59	52.00	50.70	53.30	**8.70**	6.75	10.65	-0.81	-6.25	4.63
	F	134	*1*	74.30	72.03	76.45	1.41	-1.74	4.56	78.50	77.60	79.40	**4.30**	2.80	5.80	2.89	-0.60	6.38
			5	57.87	55.05	60.63	**6.14**	2.18	10.10	62.60	61.50	63.70	**8.70**	6.96	10.44	2.56	-1.77	6.89
			10	50.06	45.77	54.34	4.76	-0.69	10.21	50.90	49.60	52.30	**12.30**	10.24	14.36	**7.54**	1.71	13.37
**Stomach**	M	173	*1*	37.70	35.09	40.32	**6.01**	2.07	9.95	49.90	48.70	51.00	1.30	-0.84	3.44	**-4.71**	-9.19	-0.23
			5	18.61	16.41	20.94	**5.58**	1.99	9.17	24.00	22.90	25.10	**2.90**	1.04	4.76	-2.68	-6.72	1.36
			10	18.54	15.34	22.08	4.27	-0.35	8.89	21.20	20.10	22.50	**2.20**	0.24	4.16	-2.07	-7.09	2.95
	F	110	*1*	38.58	35.28	41.89	2.40	-2.67	7.47	49.10	47.50	50.70	1.00	-1.97	3.97	-1.40	-7.27	4.47
			5	20.61	17.70	23.70	2.17	-2.41	6.75	25.70	24.20	27.20	0.70	-1.80	3.20	-1.47	-6.69	3.75
			10	19.59	15.39	24.32	1.67	-4.19	7.53	22.10	20.60	23.80	1.70	-0.86	4.26	0.03	-6.37	6.43
**Oesophagus**	M	154	*1*	31.89	28.94	34.89	3.60	-0.70	7.90	42.20	40.70	43.60	1.40	-1.19	3.99	-2.20	-7.22	2.82
			5	9.74	7.72	12.06	0.55	-2.57	3.67	16.20	15.00	17.50	-0.70	-2.58	1.18	-1.25	-4.89	2.39
			10	9.74	7.22	12.77	-0.80	-4.44	2.84	12.70	11.40	14.00	-0.60	-2.47	1.27	0.20	-3.89	4.29
	F	75	*1*	32.36	28.25	36.56	4.49	-1.65	10.63	42.60	40.50	44.70	1.10	-2.81	5.01	-3.39	-10.67	3.89
			5	11.87	8.91	15.33	-0.91	-5.45	3.63	18.50	16.80	20.30	-1.50	-4.31	1.31	-0.59	-5.93	4.75
			10	10.23	6.19	15.65	-2.86	-8.46	2.74	14.10	12.40	15.90	-1.00	-3.70	1.70	1.86	-4.36	8.08

Cancer site	Sex	NZ annual deaths (2008)	Year	RSR NZ (2000-05)	95%CI of RSR NZ (2000-05)		Imp NZ (2000-05 to 2006-10)	95%CI of Imp NZ (2000-05 to 2006-10)		RSR AUS (2000-05)	95%CI of RSR AUS (2000-05)		Imp AUS (2000-05 to 2006-10)	95%CI of Imp AUS (2000-05 to 2006-10)		Difference (Imp AUS - Imp NZ)	95%CI of Difference	
**Brain**	M	109	*1*	35.50	32.42	38.59	**6.13**	1.45	10.81	43.20	41.70	44.60	**3.80**	1.17	6.43	-2.33	-7.70	3.04
			5	17.82	15.42	20.38	0.68	-3.13	4.49	18.70	17.60	19.90	1.70	-0.19	3.59	1.02	-3.23	5.27
			10	14.00	11.64	16.58	1.01	-2.64	4.66	14.20	13.20	15.30	1.60	-0.11	3.31	0.59	-3.44	4.62
	F	98	*1*	34.97	31.27	38.70	**6.79**	1.28	12.30	40.80	39.10	42.50	**6.00**	2.85	9.15	-0.79	-7.14	5.56
			5	20.92	17.81	24.21	2.14	-2.63	6.91	20.30	19.00	21.70	**3.60**	1.23	5.97	1.46	-3.87	6.79
			10	16.23	12.93	19.87	1.95	-2.85	6.75	16.40	15.10	17.70	**3.50**	1.24	5.76	1.55	-3.76	6.86
**Bladder**	M	134	*1*	85.35	83.61	86.97	**-12.85**	-16.05	-9.65	83.00	82.20	83.80	-1.30	-2.83	0.23	**11.55**	8.01	15.09
			5	70.26	67.64	72.83	**-17.16**	-21.29	-13.03	62.40	61.20	63.60	**-2.40**	-4.40	-0.40	**14.76**	10.17	19.35
			10	66.35	62.03	70.67	**-17.49**	-23.06	-11.92	54.60	53.10	56.10	-2.20	-4.47	0.07	**15.29**	9.28	21.30
	F	66	*1*	77.66	74.45	80.59	**-16.66**	-22.21	-11.11	72.60	71.00	74.20	-2.70	-5.75	0.35	**13.96**	7.63	20.29
			5	64.38	60.19	68.43	**-18.64**	-25.15	-12.13	54.00	52.10	56.00	**-4.40**	-7.73	-1.07	**14.24**	6.93	21.55
			10	65.99	59.18	72.73	**-21.47**	-30.18	-12.76	47.70	45.50	50.00	**-3.90**	-7.45	-0.35	**17.57**	8.16	26.98
**Liver**	M	124	*1*	21.32	18.33	24.48	**9.65**	5.19	14.11	34.10	32.50	35.60	**4.00**	1.31	6.69	**-5.65**	-10.86	-0.44
			5	8.00	6.08	10.26	**4.91**	1.59	8.23	12.20	11.00	13.30	**3.30**	1.49	5.11	-1.61	-5.39	2.17
			10	7.70	5.68	10.13	**3.81**	0.27	7.35	9.30	8.20	10.50	**2.00**	0.23	3.77	-1.81	-5.77	2.15
	F	66	*1*	23.81	19.31	28.61	-1.42	-7.61	4.77	31.60	29.30	34.00	**5.50**	1.33	9.67	6.92	-0.54	14.38
			5	12.69	9.11	16.92	0.45	-4.76	5.66	12.50	10.70	14.40	2.90	-0.05	5.85	2.45	-3.54	8.44
			10	8.56	5.15	13.09	0.18	-5.12	5.48	9.60	7.90	11.40	2.70	-0.15	5.55	2.52	-3.50	8.54
**Ovary**	F	184	*1*	70.13	67.80	72.34	**-5.20**	-8.66	-1.74	73.70	72.60	74.70	**2.80**	0.93	4.67	**8.00**	4.07	11.93
			5	45.56	43.03	48.07	**-9.71**	-13.37	-6.05	40.20	39.00	41.50	**3.10**	1.11	5.09	**12.81**	8.65	16.97
			10	41.45	38.43	44.48	**-10.47**	-14.46	-6.48	32.70	31.50	34.00	1.30	-0.65	3.25	**11.77**	7.33	16.21
**Kidney**	M	98	*1*	72.14	69.43	74.68	**6.52**	3.02	10.02	81.70	80.80	82.70	**2.90**	1.37	4.43	-3.62	-7.44	0.20
			5	57.50	54.17	60.75	**4.73**	0.18	9.28	66.10	64.80	67.40	**5.50**	3.55	7.45	0.77	-4.18	5.72
			10	50.89	45.34	56.47	3.79	-3.05	10.63	57.80	56.20	59.40	**6.10**	3.77	8.43	2.31	-4.92	9.54
	F	67	*1*	72.02	68.52	75.22	**6.62**	2.04	11.20	79.00	77.70	80.20	**4.90**	2.79	7.01	-1.72	-6.76	3.32
			5	57.00	52.90	60.96	**10.42**	4.72	16.12	66.40	64.70	68.00	**6.10**	3.51	8.69	-4.32	-10.58	1.94
			10	52.35	46.74	57.88	**11.88**	4.50	19.26	58.80	56.80	60.70	**8.00**	5.02	10.98	-3.88	-11.84	4.08
**Myeloma**	M	96	*1*	72.41	68.95	75.62	3.68	-1.04	8.40	74.80	73.30	76.30	**4.10**	1.52	6.68	0.42	-4.96	5.80
			5	35.52	31.59	39.54	**7.96**	2.13	13.79	36.30	34.50	38.10	**7.60**	4.76	10.44	-0.36	-6.85	6.13
			10	15.72	10.57	22.02	**10.47**	3.22	17.72	18.20	16.50	20.00	**5.80**	3.14	8.46	-4.67	-12.39	3.05
	F	68	*1*	72.49	68.55	76.08	3.36	-1.97	8.69	74.20	72.40	75.80	**3.30**	0.36	6.24	-0.06	-6.15	6.03
			5	34.47	30.06	39.00	4.56	-1.89	11.01	36.40	34.40	38.40	**6.40**	3.28	9.52	1.84	-5.33	9.01
			10	21.05	14.77	28.39	3.56	-4.71	11.83	18.50	16.70	20.50	**5.90**	2.99	8.81	2.34	-6.43	11.11
